# Predicted early fusion intermediates in the spike of ACE2‐utilising bat coronavirus unveil broad‐spectrum antiviral mechanisms

**DOI:** 10.1002/ctm2.70459

**Published:** 2025-09-08

**Authors:** Lujia Sun, Zhimin Liu, Lixiao Xing, Xiaoxing Liang, Lu Lu, Shibo Jiang, Lei Sun, Xinling Wang

**Affiliations:** ^1^ Shanghai Institute of Infectious Disease and Biosecurity Key Laboratory of Medical Molecular Virology (MOE/NHC/CAMS) School of Basic Medical Sciences, Institute of Biomedical Sciences Fudan University Shanghai China; ^2^ Shanghai Fifth People's Hospital Fudan University Shanghai China

1

A recent groundbreaking study by Xing et al. published in *Cell* has successfully captured the early fusion intermediate conformation (E‐FIC) of severe acute respiratory syndrome coronavirus 2 (SARS‑CoV‑2) spike (S) protein induced by ACE2.^1^ In this conformation, the heptad repeat 1 (HR1) domain of the S2 subunit has ejected, while the S1 subunit carrying the receptor binding domain (RBD) still binds to S2. This intermediate conformation establishes a distinctive therapeutic window wherein the spatial separation between the RBD and HR1 domain enables concurrent engagement by a dual‐target inhibitor, AL5E (targeting both RBD and HR1), which sterically blocks conformational transitions essential for fusion pore expansion, thereby inhibiting viral fusion and entry and potentially inducing viral inactivation.^1^, ^2^

While elucidation of the SARS‐CoV‐2 E‐FIC has addressed a significant knowledge gap,^3^ the fusion intermediate states for most coronaviruses remain uncharacterized. Recent evidence indicates that several MERS‐related coronaviruses (MERSr‐CoVs), such as NeoCoV,^4^ MOW15‐22^5^ and HKU5,^6^ utilise bat or non‐bat ACE2 as a functional receptor. Notably, among these, BtHKU5‐CoV‐2‐441(BtHKU5‐CoV‐2) can utilise human ACE2 (hACE2) to mediate host cell infection.^7^ Furthermore, NeoCoV S protein containing a T510F mutation has acquired the capacity to bind the hACE2 receptor, representing a potential threat of zoonotic spillover.^4^ Consequently, investigating the fusion mechanisms of these MERSr‐CoVs and developing broad‐spectrum membrane fusion inhibitors that exploit such mechanisms should be considered an imperative for mitigating the potential risks of cross‐species transmission.

Structural analysis of the SARS‐CoV‐2 E‐FIC revealed that an amino acid sequence, designated as one intermediate loop (IL)—IL770, engages the HR1 domain within the E‐FIC S2 subunit (Figure 1A). Notably, the S2' protease cleavage site also resides within IL770. Consequently, elucidating variations in IL770 across coronaviruses holds essential implications for understanding both protease accessibility and the regulation of membrane fusion efficiency in the E‐FIC S2 context. To explore the fusion mechanisms of such hACE2‐using MERSr‐CoVs, we first predicted the three‐dimensional structures of BtHKU5‐CoV‐2 and NeoCoV S2 in E‐FIC using SWISS‐MODEL homology modelling.^8^ The overall structures of the S2 in E‐FIC from BtHKU5‐CoV‐2 and NeoCoV resemble that of SARS‐CoV‐2, in which HR1, CH and part of the FP form a long central three‐stranded coiled coil (Figure 1B). This coiled‐coil is stabilised by a three‐helix segment (3H) and an IL replaced by HR2 in the post‐fusion structure.^9^ Consequently, the characteristics of IL, especially at the site replaced by HR2, may have potential implications for membrane fusion and the related effects of such fusion inhibitors as EK1.^10^ However, IL from BtHKU5‐CoV‐2 (71 amino acids, residues 815–885) and NeoCoV (66 amino acids, residues 829–894) contain more amino acids compared to that of SARS‐CoV‐2 (62 amino acids, residues 770–831), and, structurally, these excess amino acids are mainly enriched in the region near the S2'‐site (Figure 1C and Figure S1). Structural alignment further revealed conserved spatial positioning of the S2' protease cleavage sites in BtHKU5‐CoV‐2 and NeoCoV relative to SARS‐CoV‐2 (Figure 1D). Key residues mediating HR1 contacts in SARS‐CoV‐2 exhibit divergence, such as K795, D796, F797, F802 and K825, which correspond to G841, L842, N843, L848 and K879 in BtHKU5‐CoV‐2 and T854, G855, F856, L862 and S888 in NeoCoV, respectively. These residue substitutions may attenuate the interaction between IL and HR1, thereby increasing the conformational dynamics of IL.

**FIGURE 1:**
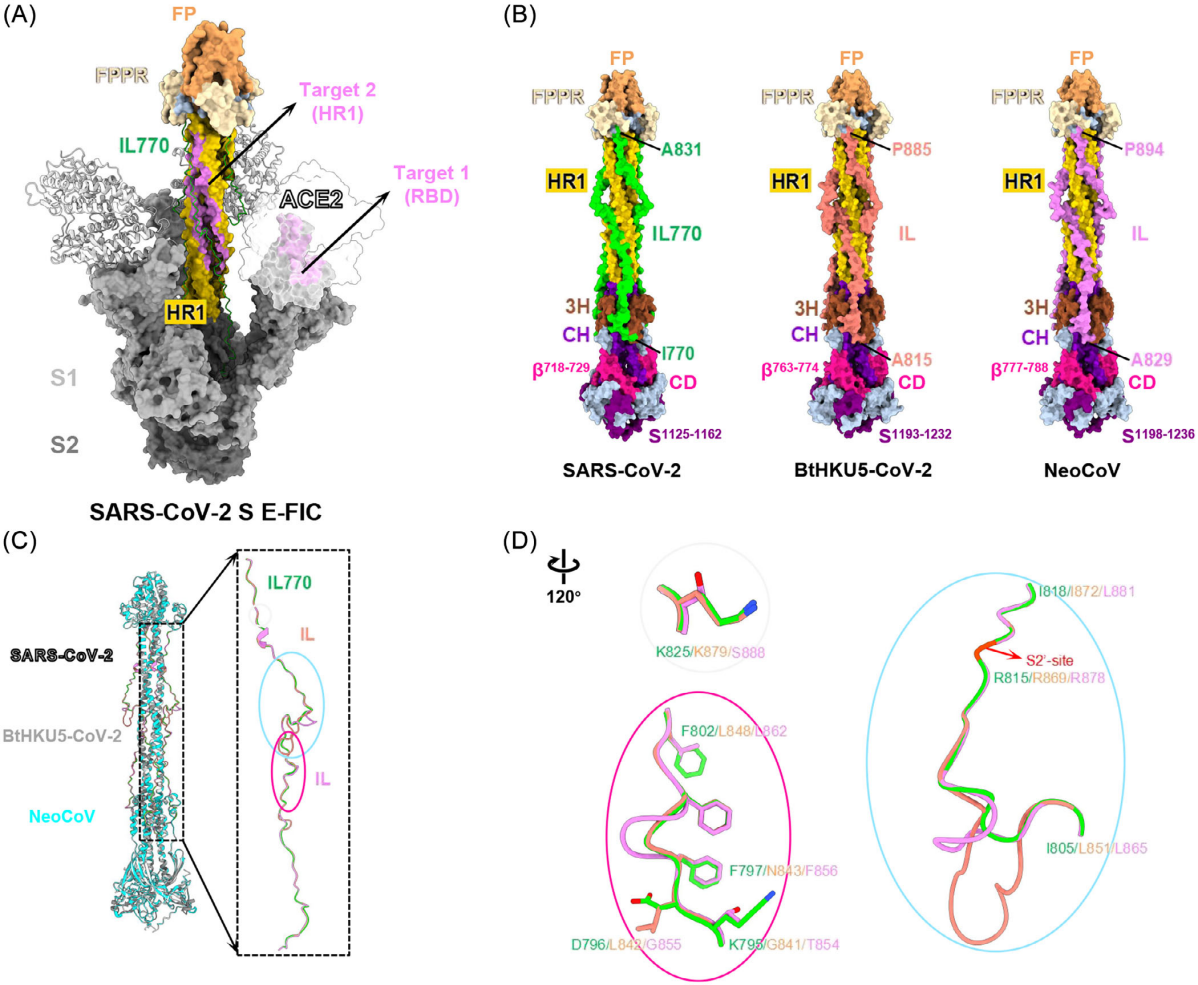
Prediction of the early fusion intermediate conformation (E‐FIC) of BtHKU5‐CoV‐2 and NeoCoV S2 subunits. (A) Two binding targets (violet) of ACE2 and HR2 in E‐FIC (PDB: 8Z7P) induced by ACE2 (white). The S1 and S2 subunits are labelled in dark and dim grey, respectively. The IL770, FPPR, FP and HR1 are in lime, wheat, sandy brown, and gold, respectively. (B) Surface representation of the SARS‐CoV‐2, BtHKU5‐CoV‐2 and NeoCoV S2 subunits in E‐FIC. The model of SARS‐CoV‐2 S2 was derived from the locally refined cryo‐EM structure (PDB: 8Z7G) uploaded to the PDB database, whereas the models of BtHKU5‐CoV‐2 and NeoCoV S2 were generated by using SWISS‐MODEL Homology Modelling. (C) Structural comparison of the E‐FIC S2 from SARS‐CoV‐2 (white), BtHKU5‐CoV‐2 (grey), and NeoCov (cyan) spike with IL in lime, salmon, and violet, respectively. ILs are highlighted. (D) Magnified views of the ILs in (C) are shown with a 120° rotation. 3H, three‐helix segment; CD, connector domain; CH, central helix; FP, fusion peptide; FPPR, fusion peptide proximal region; HR1, heptad repeat 1; HR2, heptad repeat 2; IL, intermediate loop.

Given the high conservation of the HR1 domain across coronaviruses and the structural plasticity of IL, broad‐spectrum inhibitors, such as EK1C4, could efficiently suppress pseudotyped BtHKU5‐CoV‐2 entry.^7^ The previously noted dual‐target inhibitor AL5E, which is based on the SARS‐CoV‐2 E‐FIC structure and engineered by linking EK1 with ACE2, showed potent inhibition against ACE2‐utilising coronaviruses.^1^ In this study, we further examined the inhibitory effect of AL5E on two hACE2‐using MERSr‐CoVs, BtHKU5‐CoV‐2 and NeoCoV (T510F) (Figure 2). Strikingly, AL5E exhibited nanomolar inhibitory potency against pseudotyped BtHKU5‐CoV‐2 (IC_50 _= 8 nM), representing a 28‐fold improvement over EK1 and a 220‐fold enhancement relative to hACE2. In contrast, AL5E showed only micromolar‐level inhibition against pseudotyped NeoCoV (T510F) (IC_50 _= 0.9 µM) with no evidence of significant improvement in potency compared to that of either EK1 or ACE2.

**FIGURE 2:**
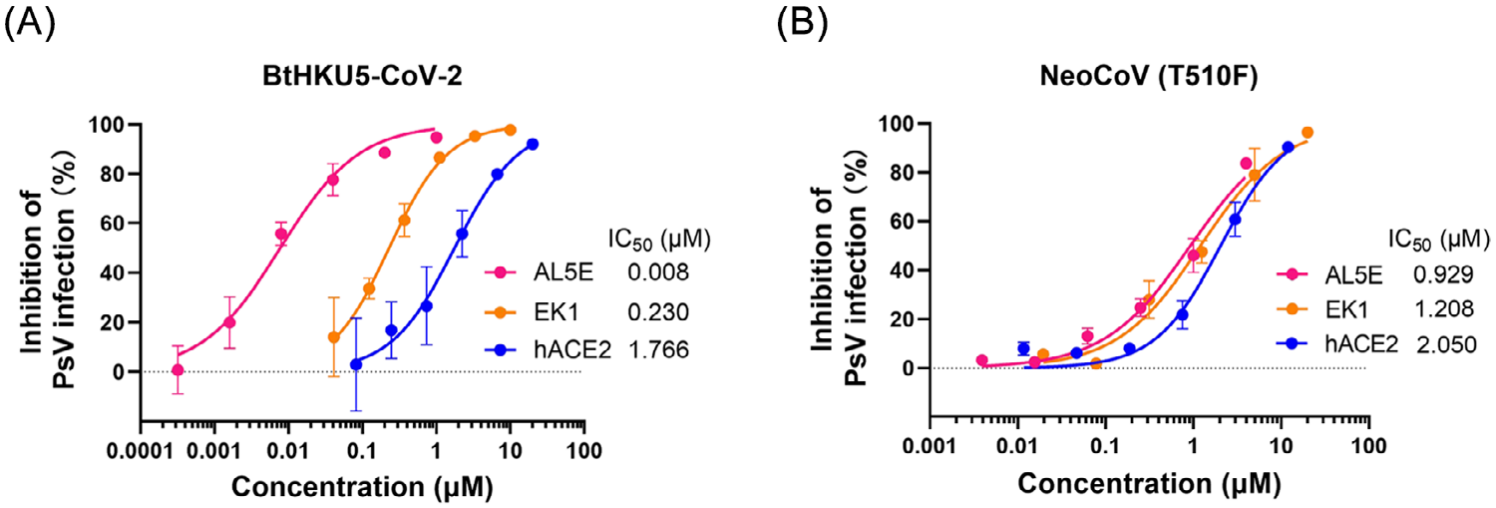
Inhibitory activity of AL5E against pseudotyped BtHKU5‐CoV‐2 and NeoCoV (T510F). (A) Inhibitory activity of AL5E against pseudotyped BtHKU5‐CoV‐2. (B) Inhibitory activity of AL5E against pseudotyped NeoCoV (T510F).

The differential efficacy of AL5E against BtHKU5‐CoV‐2 versus NeoCoV (T510F) arises from distinct conformational landscapes in their spike proteins. To explain, non‐conservative substitutions in BtHKU5‐CoV‐2 (e.g., G841, L842, N843 and L848) destabilise the IL‐HR1 interface, which results in increasing both IL conformational flexibility and HR1 solvent accessibility. This structural configuration enables AL5E to engage with targets HR1 and RBD simultaneously and establish synergistic inhibition through spatial proximity. Conversely, while NeoCoV (T510F) boosts hACE2 binding affinity, it may also induce long‐range allosteric reorganisation of the spike trimer. This rearrangement could displace HR1 beyond the optimal binding radius of dual‐functional AL5E. Consequently, even though substitutions in NeoCoV (e.g., T854, G855, L862 and S888 in the S2 subunit) may destabilize the IL‐HR1 interface to, in turn, increase IL conformational flexibility, similar to the efficacy of AL5E against BtHKU5‐CoV‐2, the retention of a hydrophobic amino acid residue, F856, may allow it to interact closely with the hydrophobic patch formed by HR1. An inverse result occurs whereby the IL of NeoCoV is stabilised, and potency is attenuated, as noted above, in comparison to BtHKU5‐CoV‐2. This mechanistic divergence explains the differential inhibitory potency of EK1/AL5E against the two MERSr‐CoVs. It also explains why the differential inhibitory activities of AL5E against distinct MERSr‐CoVs must be considered in the future design and engineering of fusion inhibitors.

Meanwhile, we found that the amplified conformational flexibility of IL facilitates EK1 binding to HR1, while concurrently promoting six‐helix bundle (6‐HB) formation between HR1 and HR2 during post‐fusion. This conformational adaptability may be the key mechanistic determinant in the balance between infection efficiency and host adaptability in the cross‐species transmission of coronavirus. With this in mind, inhibitors targeting the E‐FIC process should be rationally optimised in combination with virus‐specific conformational features.^2^ For instance, incorporating an extended flexible linker into AL5E could enable dynamic adaptation to spatial distance variations between the RBD and HR1 domains across diverse coronaviruses. Alternatively, developing small molecules or peptides capable of conformation‐specific recognition of the accessible HR1 domain could transcend design limitations rooted solely in sequence conservation.

In sum, this study reveals the diversity in membrane fusion mechanisms between two hACE2‐using MERSr‐CoVs, BtHKU5‐CoV‐2 and NeoCoV (T510F), by integrating structural modelling and functional analysis focused on IL. Furthermore, the differential inhibitory activities of AL5E against distinct MERSr‐CoVs have profound implications for the design and engineering of de novo E‐FIC‐specific fusion inhibitors. As implied in this work, broad‐spectrum antiviral strategies need to break through the limitations of static target recognition and turn, instead, to precise intervention in the dynamic conformational landscape of the fusion process with the aim of providing innovative solutions to the threat of emerging coronaviruses.

## AUTHOR CONTRIBUTIONS

**Conceptualisation**: Shibo Jiang, Lei Sun, Xinling Wang and Lu Lu; **Data curation**: Lujia Sun, Lixiao Xing and Xiaoxing Liang; **Funding acquisition**: Shibo Jiang, Xinling Wang, Lei Sun and Lu Lu; **Methodology and software**: Zhimin Liu; **Visualisation**: Lujia Sun, Zhimin Liu and Lixiao Xing; **Formal analysis and writing—original draft**: Lujia Sun and Zhimin Liu; **Writing—review & editing**: Lu Lu, Shibo Jiang, Lei Sun and Xinling Wang. All authors have read and approved the manuscript.

## CONFLICT OF INTEREST STATEMENT

Lixiao Xing, Lu Lu, Shibo Jiang and Xinling Wang are inventors in the patent related to AL5E. This relationship did not influence the study. Other authors declare no conflict of interest.

## ETHICS STATEMENT

This article does not contain any research involving humans or animals.

## Supporting information



Supporting Information
