# Supplementary material for: Predicted early fusion intermediates in the spike of ACE2‐utilising bat coronavirus unveil broad‐spectrum antiviral mechanisms
Source: Clin Transl Med. 2025 Sep 8;15(9):e70459. doi: 10.1002/ctm2.70459 (PMC12415440; doi:10.1002/ctm2.70459)
Supplement: Supplementary file 1 — Supporting Information [file CTM2-15-e70459-s001.docx]

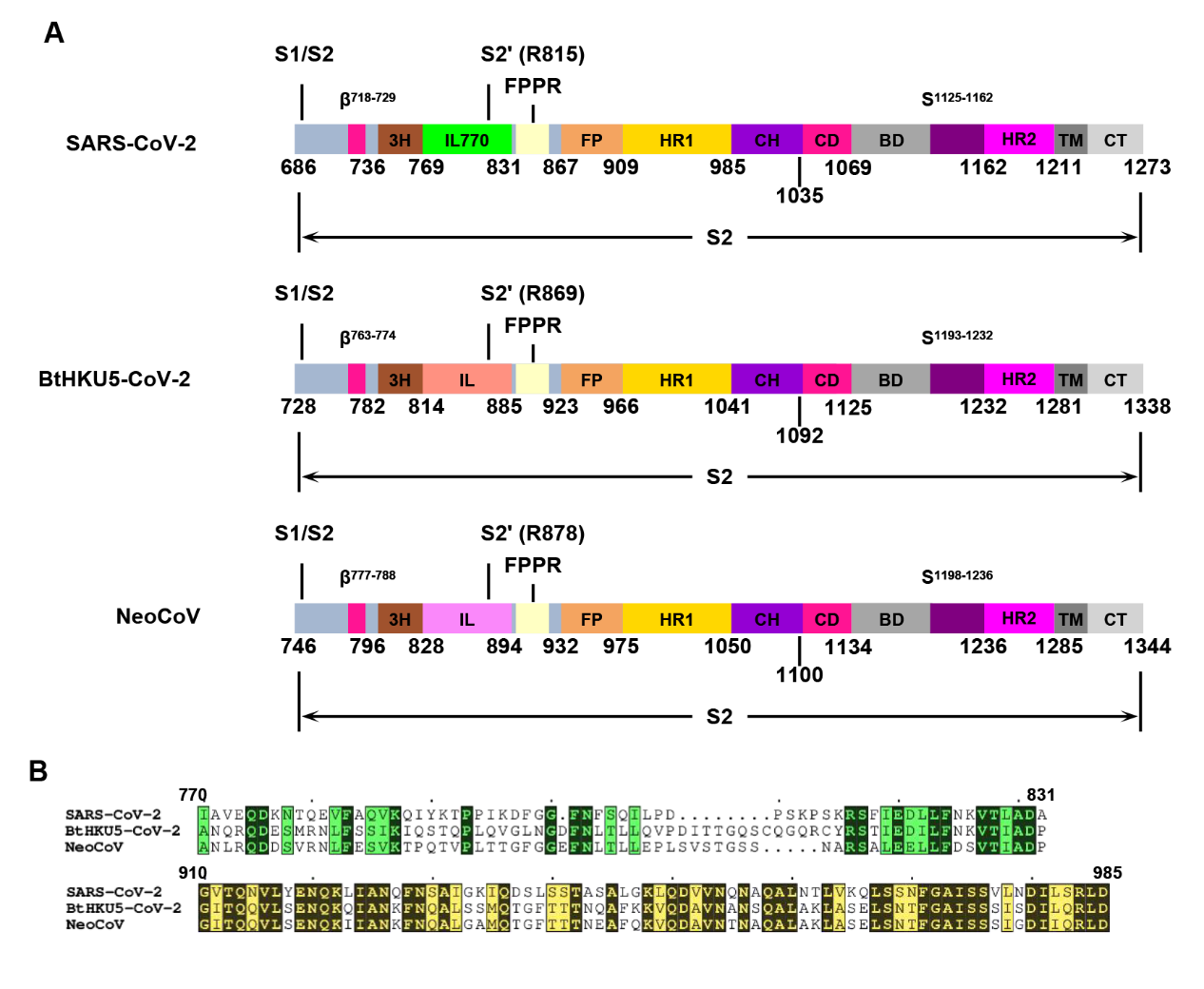


**Supplementary Figure 1.** (A) Schematic of SARS-CoV-2 S2 fragment, which contains the following segments: S1/S2, S1/S2 cleavage site; β^718–729^; 3H, three-helix segment; IL, intermediate loop; FPPR, fusion peptide proximal region; FP, fusion peptide; HR1, heptad repeat 1; CH, central helix; CD, connector domain; BD, basal domain; segment S^1,125-1,162^; HR2, heptad repeat 2; TM, transmembrane anchor; CT, cytoplasmic tail; and inverted triangles for glycans in S2. (B) Sequence alignments of IL (top) and HR1 (bottom) domains among coronaviruses were generated using MEGA and rendered with ESPript.
